# Influence of surface features on the perception of nonadjacent musical phrases

**DOI:** 10.1177/10298649221148681

**Published:** 2023-02-03

**Authors:** Joanna Spyra, Matthew Woolhouse

**Affiliations:** McMaster University, Canada

**Keywords:** figuration, rhythm, memory, nonadjacency, harmony

## Abstract

Although temporally nonadjacent key relationships (e.g., Key X →Key Y→ Key X) are ubiquitous within tonal music, the full extent to which they are perceived is uncertain. Previous research suggests that memory for an initial key remains active up to 20 s after modulation; however, homophonic textures were used in these studies, leaving open the possibility that surface features such as figuration may contribute to nonadjacency effects. In two experiments, we investigated this issue by measuring goodness of completion ratings for stimuli in which musical surface features were manipulated. Two types of surface feature were tested: figuration and activity (total number of notes per stimulus). Stimuli were composed of three parts: (1) nonadjacent section (in either the same or a different key to the probe); (2) intervening section (in a different key to the probe); and (3) probe (a cadence in either the same or different key as the nonadjacent section). In Experiment 1, we tested whether the presence of surface features resulted in higher goodness of completion ratings for the probe; in Experiment 2, we manipulated nonadjacent key relationships to ascertain the effect of surface features on global perception of key. Results showed that figuration and activity contributed to goodness of completion ratings, particularly in stimuli where these features matched each other in the nonadjacent sections. Moreover, the presence of surface features strengthened the perceived relationships between the keys of nonadjacent sections, thereby appearing to contribute to the global perception of phrase. In sum, although from an analytical perspective surface features are often considered to be less important hierarchically, our results indicate that they contribute significantly to the perception of nonadjacent key relationships.

In the hands (and minds) of accomplished composers, notes, chords, phrases, entire sections, and even movements can refer to each other across time (Laitz, 2012, pp. 371–384). For example, the basis of sonata form, arguably the major structural achievement of music from the common practice period (Piston, 1941), is predicated on this notion (Stravinsky, 1970, p. 41). However, the extent to which listeners retain nonadjacent relationships in memory, and the elements of music that enhance or diminish the effect of musical cross-referencing, so to speak, are poorly understood. To date, research has investigated listeners’ perceptions of relationships between keys over durations of varying lengths (Farbood, 2016; Woolhouse et al., 2016) and has asked if the decay of memory for a key is due simply to the passage of time or the result of interference from distracting information such as chords in a new key (Spyra et al., 2021; see also Akiva-Kabiri et al., 2009). In two experiments reported here, we investigated the importance of surface features for maintaining listeners’ sense that nonadjacent sections of music separated by modulations are nevertheless connected perceptually. Surface features include melodic and rhythmic decorations, frequently referred to as musical embellishments, of an underlying harmonic framework, a notion that is central to Schenkerian analysis, for example, (Pankhurst, 2008, p. 19). For additional comments on surface features, see also Schellenberg et al. (2014).

Cook (1987) conducted the first study of the perception of large-scale tonal structures. He used as stimuli two contrasting versions of short excerpts ranging between 30 s and 6 min, from works for piano dating from the Classical and Romantic eras; the first excerpt was in a single key, while the second included a modulation from one key to another. Participants were asked to listen to both versions of the same excerpts and make preference judgments based on characteristics of the music including expressiveness, coherence, pleasure, and sense of completion. Preferences differed significantly only for the two shortest excerpts (30 s and 1 min), suggesting that participants were unable to make judgments on excerpts lasting more than approximately 1 min. Cook’s (1987) study provided a foundation for subsequent research on the role of memory in music perception but did not distinguish between the effects of local and global influences.^1^ For this reason, it is not possible to say which elements of music are processed in working memory (see Snyder, 2000, pp. 3–15, 47–53) and, consequently, how long it took for the memory of these elements to decay or become degraded.

Using a nonadjacent key paradigm, Woolhouse et al. (2016) investigated the influence of one harmonic region on a subsequent, temporally nonadjacent harmonic region, that is, listeners’ ability to form global percepts of harmony. By comparing goodness of completion ratings of probe cadences from two matching stimuli with key structures X-Y-X and Y-Y-X, the global effect of the first X on the last X could be isolated from the local effect of Y-X. For clarity, X and Y represent the key of each section and the subscripts _ns_ (nonadjacent section), _is_ (intervening section), and _pc_ (probe cadence) represent its position within the stimulus; see Figure 1.

**Figure 1.**
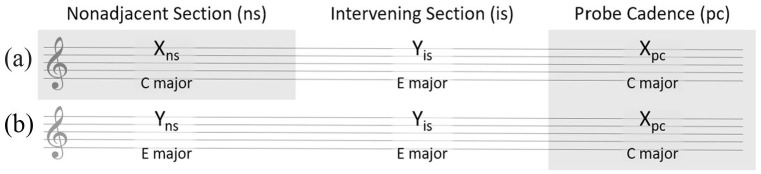
Examples of nonadjacent key relationships in which (a) the nonadjacent section and the probe cadence or (b) the nonadjacent and intervening sections are in the same key.

Woolhouse et al. (2016) found a significant effect of X_ns_ on the perception of X_pc_ and, furthermore, built on the findings of Cook (1987) by investigating the precise point at which participants were no longer able to perceive relationships between them. By manipulating the duration of Y_is_, Woolhouse et al. were able to show that X_ns_ ceased to influence the stimuli-closing probe cadence after 11 s. This was true for both musicians and nonmusicians with relative pitch.

Farbood (2016) obtained similar findings in a study extending Woolhouse et al.’s (2016) research. Instead of rating a single closing probe for goodness of completion, participants rated the tension they experienced continuously as they listened to the stimuli. The rationale was that tension would spike at every occurrence of a novelty such as a modulation to a new key. This allowed the researcher to compare the amounts of tension experienced during the first (i.e., X_ns_ to Y_is_) and second modulations (i.e., Y_is_ to X_pc_). If the second modulation felt like a return to the original key, the spike in tension would decay rapidly. Results did, indeed, show a decreasing tension slope as the duration of the intervening key increased, but not beyond 20 s.

The studies outlined above used predominantly homophonic textures in their stimuli (such as quarter-note chords or repeating arpeggios), and while these were thus harmonically coherent, they were arguably less realistic than stimuli containing surface features such as melodic figuration or varied rhythmic patterns. In contrast, we suggest that surface features may strengthen the perception of connectedness between phrases and therefore play a critical role in establishing and maintaining relationships between nonadjacent harmonies. Indeed, McAdams (1989) stated that an important criterion for perceiving structures is that deep changes should be reflected in the musical surface. In other words, if it were not for the musical surface, the perception of large-scale structures might be relatively weak. Furthermore, listeners may be more sensitive to surface features than they are to deep structures (Deliège et al., 1996) and therefore use these features to cue their memory for larger structures (see also Granot & Jacoby, 2011; Karno & Konečni, 1992). In the two experiments described below, we aimed to explore this issue by manipulating a variety of surface features and expand the research of Woolhouse et al. (2016) and Farbood (2016) by including not only homophony in our stimuli but also the musical textures found in real-world music.

Referred to as either *melodic* or *rhythmic*, figuration (*figurare* in Latin meaning shape or form) is the elaboration of a harmonic progression through the addition or rhythmic displacement of notes (Aldwell et al., 2010). *Melodic figuration* is the embellishment of an underlying melodic interval and can occur in any voice or in more than one voice simultaneously. Examples include chordal skips (a melodic leap from one chord tone to another), passing tones, and neighbor tones. Both are nonchord tones that move by step and are dissonant in the context of the surrounding harmony. Passing tones continue the melodic contour in the same direction, whereas neighbor tones return to the previous chord tone, thus reversing the direction of melodic movement. Passing tones usually occur on the offbeat; when they occur on the beat, they are referred to as accented passing tones. Neighbor tones can be upper and lower, both returning to the original chord tone from an upper or lower note as appropriate (see Figure 2). Neighbor tones that have only one stepwise connection with a chord tone are referred to as incomplete (*appoggiaturas* are examples of incomplete neighbor tones). Double neighbor tones contain a series of two incomplete neighbor tones, beginning and ending on the same chord tone (see Figure 2).

**Figure 2.**
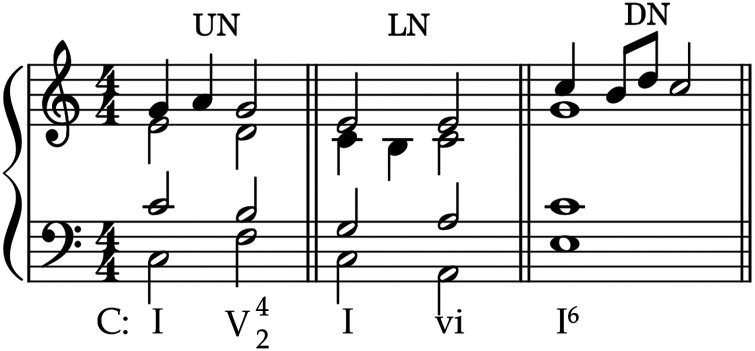
Examples of upper (UN), lower (LN), and double (DN) neighbor tones.

Like melodic figuration, *rhythmic figuration* can occur in any voice or in more than one voice simultaneously. Examples include suspensions, retardations, and anticipations. Suspensions have three features: preparation, suspension, and resolution. Briefly, a prepared chord tone (Prep) is suspended (Sus) until becoming a nonchord tone, at which point it resolves (Res) downward by stepping onto a new chord tone (see Figure 3). Retardations are upward resolving suspensions and, like suspensions, have three components: preparation, suspension, and resolution.

**Figure 3.**
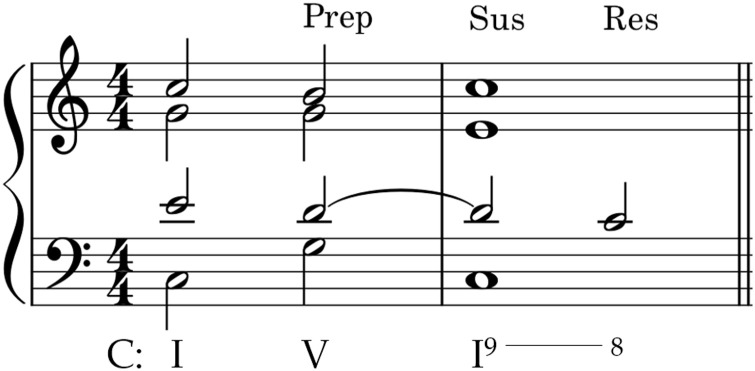
Example of a suspension in the tenor voice, indicating preparation, suspension, and resolution notes above.

Figuration in music can be found in music written prior to, during, and beyond the common practice period, and is integral to the style of a composition, be it Renaissance vocal music, Baroque keyboard suites, Classical symphonies, or Romantic lieder. For a primer on figuration, see Aldwell et al. (2010).

## Overview of study

Given the ubiquity of figuration in real-world music, our conjecture was that stimuli with surface features such as melodic and rhythmic ornamentation, as opposed to homophonic chords, would enhance the perception of structural coherence and thus lead to higher goodness of completion ratings of a closing probe. We therefore designed two experiments, both of which had three main factors and three control factors of secondary importance. The main factors were *Figuration* (the presence or absence of melodic and/or rhythmic figuration), *Activity* (the total number of notes per stimulus), and *Consistency* (whether the surface features of the outer sections of the stimulus, X_ns_ and X_pc_, matched each other; see Figure 1). The three control factors were included to ensure that our results were generalizable: *Sequence* (type of chord progression, either cycle of fifths or noncycle of fifths), *Direction* (the direction in which the intervening key modulated with respect to the probe cadence, either up or down), and *Distance* (the distance from which the intervening key modulated with respect to the probe cadence, either 2, 4, or 6 semitones). Experiment 2 had an additional main factor, *Nonadjacent key relationship*, included to investigate the influence of surface features on the perception of nonadjacent key relationships.

As discussed above, the rationale for conducting this study was to observe how surface features influence the perception of structure using real-world, specially composed musical stimuli. *Consistency* was included on the grounds that stylistic similarity and/or coherence between structural units is a salient musical percept. In summary, we hypothesized that participants would rate stimuli including *Figuration, Activity*, and *Consistency* higher for goodness of completion ratings than those with no *Figuration*, no *Activity*, and/or with inconsistent surface features.

While we explored the degree to which surface features could enhance the perception of structural cohesion in music in Experiment 1, we shifted our focus to harmonic coherence in Experiment 2 by including the factor *Nonadjacent key relationship*, seeking (like Woolhouse et al., 2016) to disentangle global and local harmonic effects by juxtaposing stimuli containing congruent nonadjacent key relationships (i.e., X to X in X_ns_-Y_is_-X_pc_) and stimuli containing incongruent nonadjacent key relationships (i.e., Z to X in Z_ns_-Y_is_-X_pc_). Here, we hypothesized that participants would have better memory for key and therefore rate stimuli with congruent nonadjacent key relationships higher for goodness of completion, particularly when the stimuli included surface features.

## Experiment 1

### Method

#### Participants

Forty-seven undergraduate university students (29 females) between the ages of 17 and 34 years (*M* = 18, *SD* = 1.7) participated in the experiment. Twenty-one had five or more years of musical training, included both self-teaching and formal lessons (*M* = 9, *SD* = 2.14). One participant failed to complete the task and was excluded from the analysis. A power analysis for a 2 × 2 × 2 repeated-measures design was conducted using the Superpower package in R v4.1.1 (Lakens & Caldwell, 2021). Data were estimated based on a previous study with a similar design and simulations were run until a power of at least 80 and an effect size of a minimum of 0.1 was reached for all main effects and at least one interaction. Simulations indicated a requirement of a minimum of 30 participants, making the exclusion of one participant acceptable. Participants were rewarded for their participation with a university course credit. Ethical approval was obtained from the McMaster Research Ethics Board (MREB #2524).

#### Apparatus

Stimuli were generated in MuseScore 2 (MuseScore Project, 2015) and exported as MIDI files using the software’s synthesized bassoon timbre. This timbre was used due to its attack rate, which was relatively fast, and sustained amplitude envelope. A balance between these two aspects of timbre was important as some stimuli included suspended notes, the perceptual clarity of which would have been lost had the timbre had a relatively fast decay (e.g., piano). Stimulus presentation was controlled through a program using the Python 3.6 programming language and its Kivy 1.9 graphic user interface package (Kivy Organization, 2016). Participants listened to the stimuli through AKG K 172 HD headphones (frequency range 18 Hz–26 kHz). Responses were entered by adjusting a 7-point slider presented through the Python program with points from 1 (*weak sense of completion*) to 7 (*strong sense of completion*). Before carrying out the task, participants adjusted the headphone volume to a comfortable level.

#### Stimuli

**Figure 4.**
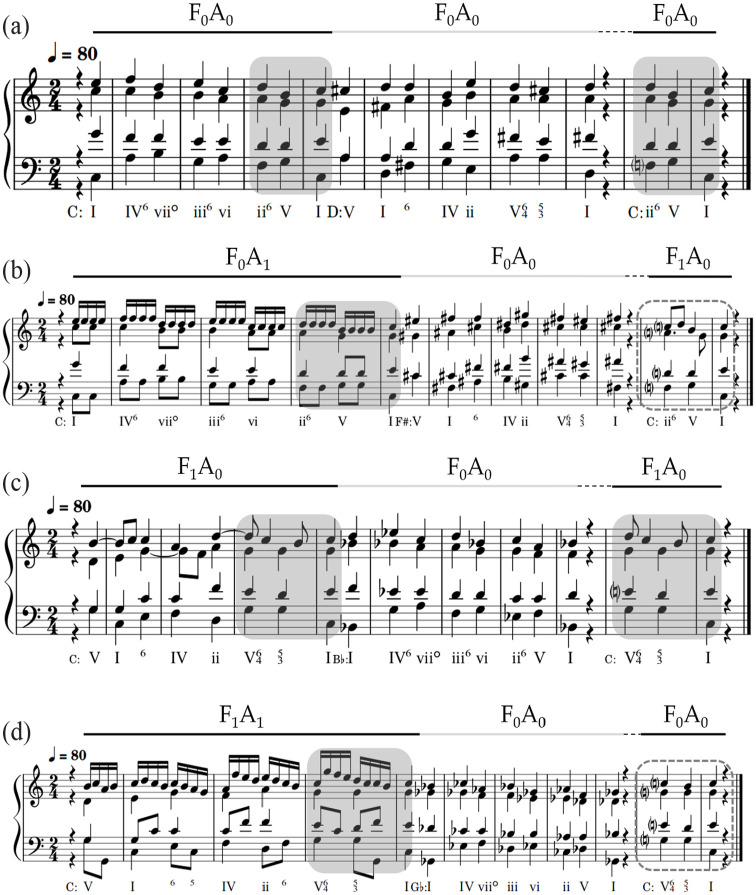
Examples of stimuli with (a) no Figuration or Activity, (b) no Figuration but with Activity present, (c) Figuration present but no Activity, and (d) both Figuration and Activity present. *Figuration* and *Activity* are denoted by the letters F and A, respectively; the subscripts _0_ and _1_ denote the presence or absence of these conditions within each factor. The highlighted gray and dashed regions indicate whether the surface features of the probe cadence were the same or different as those in the nonadjacent section.

As discussed above, the stimuli were constructed in three parts: a *nonadjacent* section (X_ns_); an *intervening* section (Y_is_); and a final *probe cadence* (X_pc_), to be rated for goodness of completion (see Figure 4). X_ns_ and Y_is_ each lasted 6 s (8 beats at 80 bpm), while X_pc_ lasted 2.25 s (3 beats). A one-beat rest separated Y_is_ from X_pc_. In total, each stimulus lasted 15 s (20 beats). X_ns_ and X_pc_ were always in the same key and randomly transposed through the 12 major keys of Western tonal-harmonic music. X_ns_ and X_pc_ (i.e., the opening and close of each stimulus) formed an over-arching tonic relationship, in much the same way that real musical phrases frequently start and end in the tonic (Laitz, 2012, p. 380). Y_is_ modulated up or down 2, 4, or 6 semitones from X_ns_ and X_pc_. All sequences conformed to music-theoretic voice-leading norms, avoiding parallel fifths and octaves.

#### Factors

**Figure 5.**
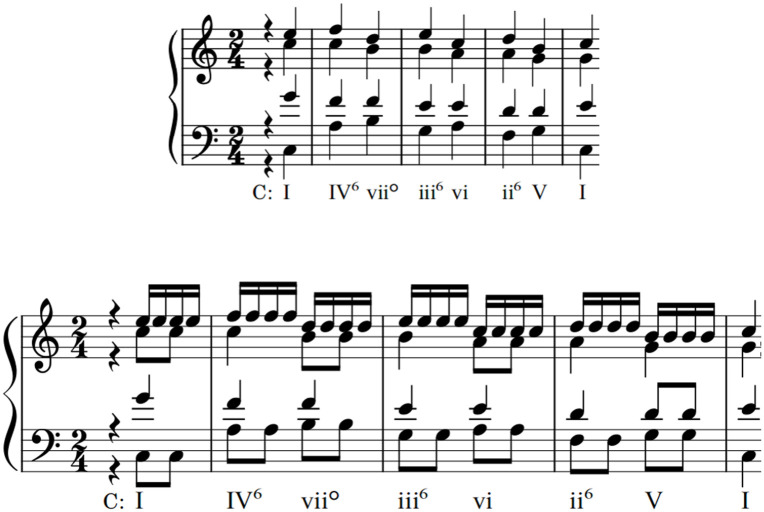
Examples of stimuli (a) with neither figuration nor rhythmic activity (F_0_A_0_) and (b) without figuration but with rhythmic activity (F_0_A_1_).

Each of the main factors had two levels. In *Figuration*, melodic and/or rhythmic figurations (e.g., passing tones and suspensions) were either present (F_1_) or absent (F_0_) in X_ns_. Similarly, in *Activity*, repeated 8th and 16th notes were either added to the melody in X_ns_ (A_1_) or omitted (A_0_). X_ns_, presented in the simplest way possible (F_0_A_0_), was composed of homophonic quarter-note chords, that is, without figuration or added activity, as shown in Figure 5a. If X_ns_ included activity but not figuration (F_0_A_1_), the topmost melodic voice was written using repeated 16th notes and additional 8th notes in the middle and low voices, as illustrated in Figure 5b.

**Figure 6.**
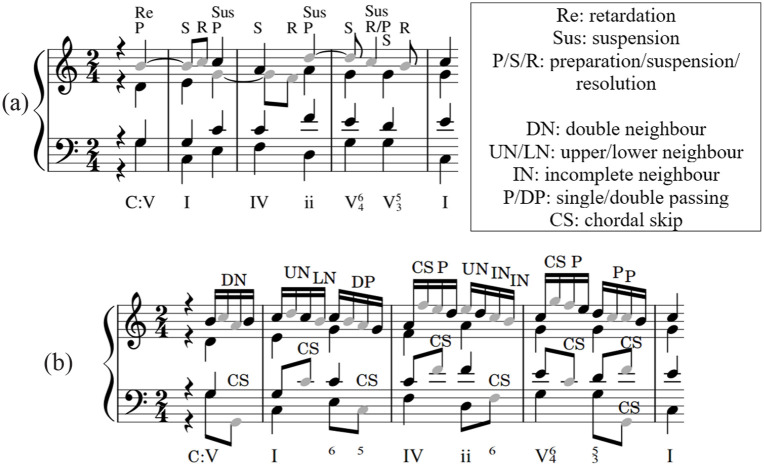
Examples of stimuli (a) with figuration but without rhythmic activity (F_1_A_0_) and (b) with figuration and rhythmic activity (F_1_A_1_).

If X_ns_ included figuration but not activity (F_1_A_0_), the upper voices contained suspensions and retardations. This provided melodic interest without adding extra notes to the sequence, thus avoiding a confound with *Activity* as shown in Figures 4c and 6a. Finally, if X_ns_ included both figuration and activity (F_1_A_1_), the top voice was written using 16th notes arranged in a melody employing passing tones, chordal skips, and neighbor tones as shown in Figures 4d and 6b. Y_is_ was always composed using a homophonic texture (F_0_A_0_).

The factor *Consistency* was used to explore the perception of the relationship between the surface features of the initial key and the probe cadence, that is, between X_ns_ and X_pc_. Surface features were either the same or different (PC_same_; PC_diff_), as shown by the shaded and dashed outlined areas in Figure 4. In the PC_diff_ condition, only complete surface feature reversals were used, as opposed to every combination of *Figuration* and *Activity*. For example, if the X_ns_ figuration combination was F_1_A_1_, the corresponding X_pc_ figuration combination was F_0_A_0_; if the X_ns_ combination was F_1_A_0_, the corresponding X_pc_ combination was F_0_A_1_; and so on.

The factors *Sequence, Direction*, and *Distance* were added as controls for generalizability and to mitigate participant fatigue and stimulus familiarity. *Sequence* refers to harmonic progression and had two levels, X_ns_ and Y_is_, which could be written using either a cycle of fifths (S_c5_: I-IV-vii°-iii-vi-ii-V-I) or a regular chord progression (S_reg_: V-I-I^6^-IV-ii-V^6^_4_-V^5^_3_-I). X_ns_ and Y_is_ always contrasted with one another in terms of the chord progression. X_pc_ was derived from the last three harmonies of X_ns_ (S_c5_: ii-V-I; S_reg_: V^6^_4_-V^5^_3_-I). *Direction* and *Distance* were used to investigate perceptions of the modulations between X_ns_ and Y_is_, which modulated up or down (*Direction*) by 2, 4, or 6 semitones (*Distance*).

#### Procedure

Prior to carrying out the task, each participant gave informed consent, completed a demographic questionnaire, and listened to two novel practice stimuli. Ninety-six stimuli were generated by combining the conditions within *Figuration*, *Activity*, *Consistency*, *Sequence*, *Direction*, and *Distance* (2 × 2 × 2 × 2 × 2 × 3 = 96). These stimuli were then presented to each participant in a randomized order and key. Following each stimulus, participants were prompted to rate the goodness of completion of the probe cadence using a Likert-type sliding scale from 1 (*not at all*) to 7 (*strong sense of completion*).

### Results

**Figure 7.**
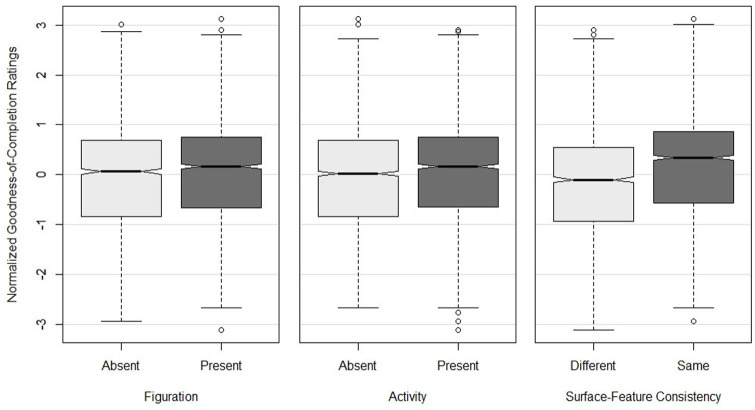
Boxplots of normalized goodness of completion ratings for main effects of *Figuration*, *Activity*, and *Consistency.* Notches represent medians.

**Figure 8.**
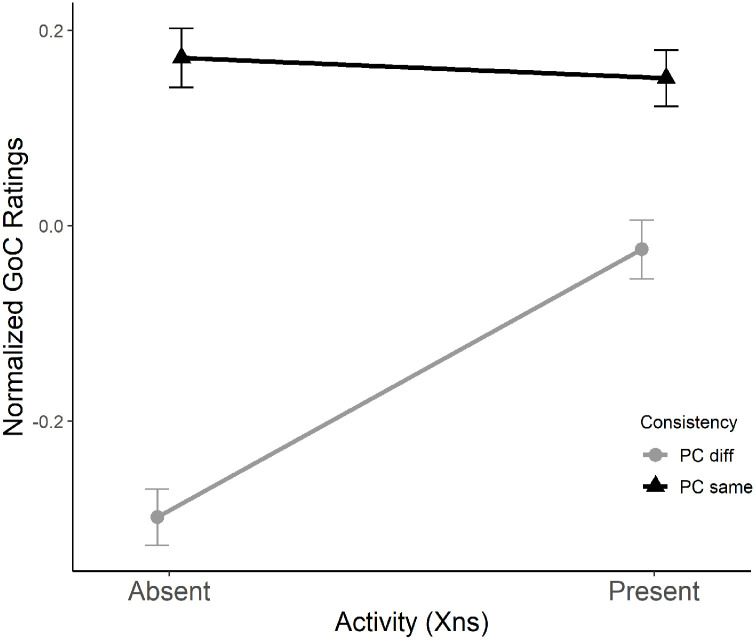
Interaction between *Activity* and *Consistency* using mean normalized goodness of completion (GoC) scores and standard error.

A 2 × 2 × 2 repeated-measures analysis of variance (ANOVA) was conducted to explore the effects of *Figuration* (two levels: F_0_ and F_1_), *Activity* (two levels: A_0_ and A_1_), and *Consistency* (two levels: PC_same_ and PC_diff_). There was a significant main effect of *Figuration* (*F*_1,45_ = 8.75, *p* = .005, $\eta_{p}^{2}$ = .163) such that stimuli including figuration were rated higher than stimuli without figuration (*M_F1_* = 0.05, *M_F0_* = −0.05). There was also a significant main effect of *Activity* (*F*_1,45_ = 22.33, *p* < .001, $\eta_{p}^{2}$ = .332) such that stimuli with more activity were rated higher than those with less activity (*M_A1_* = 0.06, *M_A0_* = −0.06). The main effect of *Consistency* was also significant (*F*_1,45_ = 41.03, *p* < .001, $\eta_{p}^{2}$ = .477): stimuli in which the surface features of X_ns_ matched the probe were rated higher than nonmatching stimuli (*M*_PCsame_ = 0.16, *M*_PCdiff_ = −0.16; see Figure 7). There was a significant interaction between *Activity* and *Consistency* (*F*_1,45_ = 7.10, *p* < .05, $\eta_{p}^{2}$ = .136) such that participants rated stimuli with matching textures more highly overall but rated nonmatching stimuli differently depending on the degree of activity in X_ns_ (PC Same: *M_A0_* = 0.17, *M_A1_* = 0.15; PC Diff: *M_A0_* = −0.30, *M_A1_* = −0.02; Figure 8).

Control factors were not included in analyses as these variables were not part of our research question and each additional variable underpowers the findings. Previous research has found consistent effects of modulation (see, e.g., Cuddy & Thompson, 1992; Spyra et al., 2021) which we have no reason to suspect would not be present in this study as well. The control factor *Sequence*, however, was added to the ANOVA in a post hoc analysis to compare the effect of musical ornamentation (i.e., surface features) to that of the harmonic progressions used. No significant effect of *Sequence* was found (*p* = .10). To further test the potential effects of musical training on the results, *Musician* (two levels: musician, nonmusician) was included as a between-group factor in the 2 × 2 × 2 repeated-measures ANOVA as described above. There was no significant interaction between *Musician* and any other main effect, suggesting that musical training may not significantly affect the ability to perceive relatively short nonadjacent relationships.

### Discussion

In Experiment 1, we explored the effects of surface features on nonadjacent musical phrases, hypothesizing that the inclusion of surface features would result in higher goodness of completion ratings of closing probe cadences. Consistent with the hypothesis, mean ratings were significantly higher for stimuli that included figuration and/or activity. Moreover, stimuli in which the surface features of the initial key matched those of the probe cadence were rated significantly higher than those with mismatched surface features, indicating the importance of stylistic consistency and/or coherence in the perception of musical form. In sum, the homophonic F_0_A_0_ condition elicited the lowest ratings (*M* = −0.09), followed by F_1_A_0_ (*M* = −0.04), F_0_A_1_ (*M* = −0.01), and the F_1_A_1_ (*M* = 0.14) condition which elicited the highest ratings. Furthermore, the difference between mean completion ratings between levels of *Consistency* was more than twice as large as those for any other factor in the experiment, highlighting the relative size of its influence (*M*_PCsame_ = 0.16, *M*_PCdiff_ = −0.16, difference score = 0.32. *Figuration* difference = 0.10, *Activity* difference = 0.12).

The significant interaction between *Activity* and *Consistency* provides further support for the findings discussed above. There were no significant effects of *Activity* if the surface features of nonadjacent sections did not match but, if they did match, the effects of *Activity* were pronounced. This suggests that it is important for the probe to be exactly the same if relationships between nonadjacent sections are to be perceived. When there is no consistency between the surface features of the two nonadjacent sections, activity in X_ns_ produces the sense that they are related, perhaps because of the listener’s familiarity with music that has such features or the expectation that activity is likely to decrease at the end of a phrase.

## Experiment 2

In Experiment 2, we targeted memory for key by adding the factor of *Nonadjacent key relationship*, which was either congruent (i.e., nonadjacent sections were in the same key) or incongruent (they were in different keys). We hypothesized that the presence of surface features would increase memory for key; congruent nonadjacent key relationships would be rated higher for goodness of completion when surface features were present.

### Methods

#### Participants

Eighty-three undergraduate university students (68 females, 33 males, 6 no responses) between the ages of 17 and 32 years (*M* = 19, *SD* = 2.3) participated in the experiment. Of these, 26 were categorized as musicians with at least 5 years of musical training (*M* = 7.6, *SD* = 2). The experiment was covered by the same ethics license as Experiment 1 and one course credit was awarded for participation. One nonmusician failed to complete the experiment and was excluded from the analysis. Given the 2 × 2 × 2 × 2 design of the experiment, a power analysis could not be performed. An appropriate *n* was therefore estimated based on Experiment 1.

#### Apparatus

Stimuli were generated in MuseScore 3 (MuseScore Project, 2015) using the software’s synthesized flute timbre. As we had selected the bassoon timbre in Experiment 1, we selected the flute timbre in Experiment 2 because of its attack rate and sustained amplitude envelope. Stimulus presentation and data collection were controlled by a program built in PsychoPy3 and PsychoJS (Peirce et al., 2019), hosted on *Pavlovia* (https://pavlovia.org/). Due to the Covid-19 pandemic, the experiment was run virtually with participants using their own personal devices and headphones.

#### Stimuli

Stimuli were similar to those in Experiment 1, including the main factors *Figuration, Activity*, and *Consistency*. As discussed previously, an additional factor was included: *Nonadjacent key relationship*, which could be either congruent or incongruent. This factor was added to target memory for key. When *Nonadjacent key relationship* was congruent, stimuli began and ended in the same key (X_ns_-Y_is_-X_pc_); when *Nonadjacent key relationship* was incongruent, the stimuli proceeded through three different keys, creating a condition that had no harmonic completion (Z_ns_-Y_is_-X_pc_). As in Experiment 1, control factors *Sequence*, *Direction*, and *Distance* were also included.

#### Procedure

Each participant completed an online demographic survey, including a consent form, before being taken to the Pavlovia platform to begin the online experiment. Instructions were provided on the platform and participants were given three practice trials to familiarize themselves with the task. Results were recorded by the PsychoPy program and stored in a csv file on the Pavlovia server.

### Results

**Figure 9.**
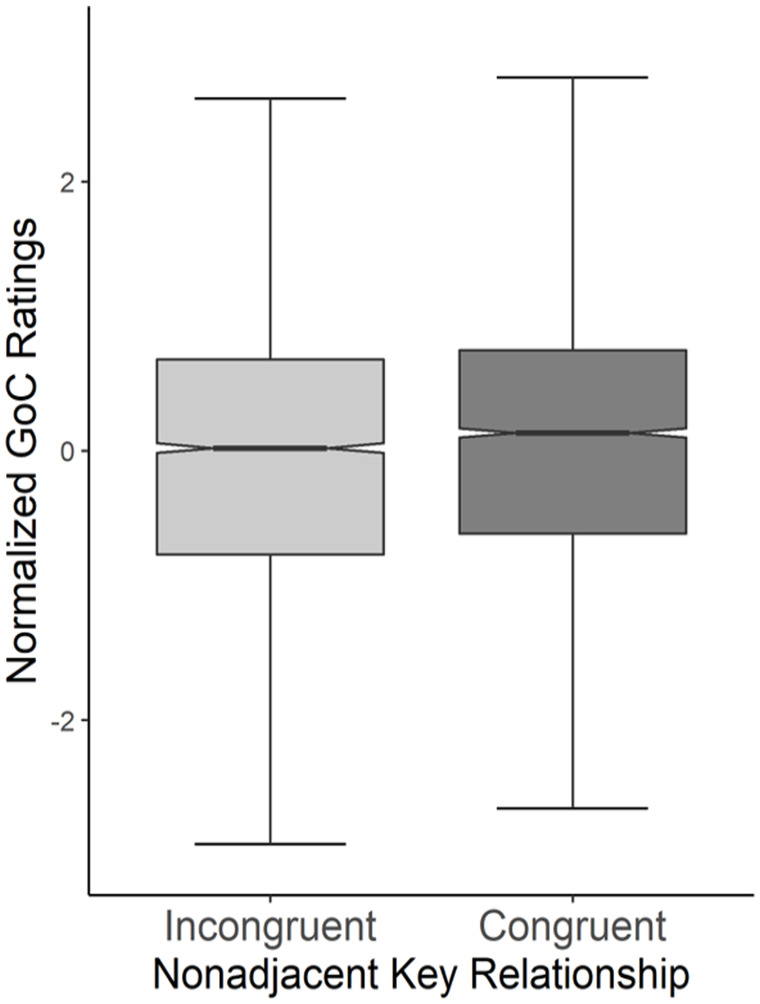
Boxplots of goodness of completion (GoC) ratings for stimuli with congruent and incongruent key relationships. Notches represent medians.

A 2 × 2 × 2 × 2 repeated-measures ANOVA was conducted with the four main factors, each with two levels: *Figuration* (present and absent), *Activity* (present and absent), *Consistency* (same and different), and *Nonadjacent key relationship* (congruent and incongruent). There was a significant main effect of *Figuration* (*F*_1,81_ = 7.57, *p* < .05, $\eta_{p}^{2}$ = .085) such that stimuli with figuration were rated higher than those without figuration (*M_F0_* = −0.03, *M_F1_* = 0.03). There was also a significant main effect of *Nonadjacent key relationship* (*F*_1,81_ = 30.21, *p* < .001, $\eta_{p}^{2}$ = .272), such that congruent relationships were rated higher than incongruent (*M_con_* = 0.07, *M_inc_* = −0.07; Figure 9). However, in contrast to the results of Experiment 1, there was no main effect of either *Activity* (*p* = .80) or *Consistency* (*p* = .23).

**Figure 10.**
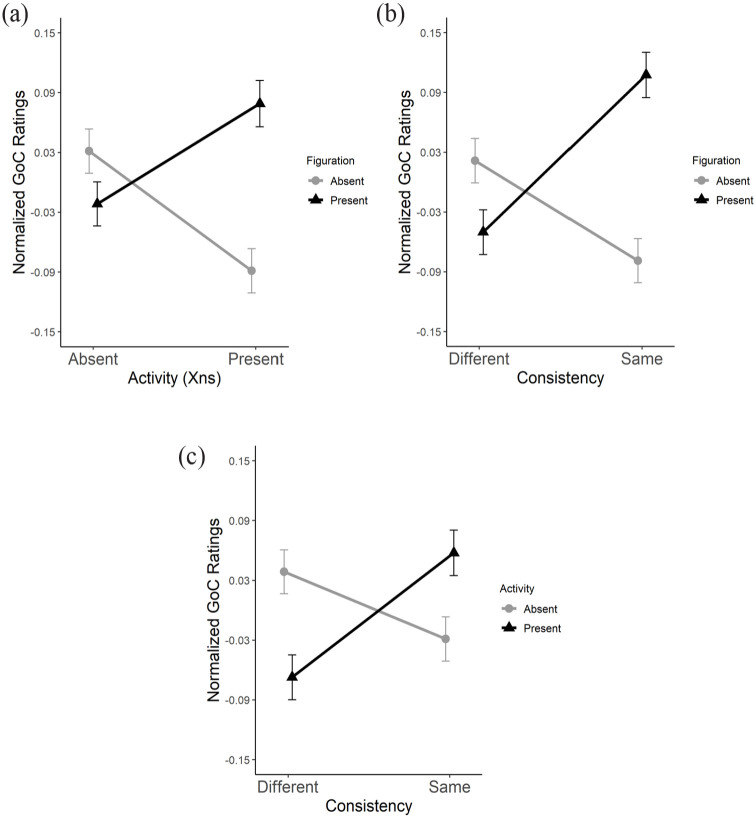
Interactions between goodness of completion (GoC) ratings for (a) *Activity*, (b) *Figuration*, and (c) *Consistency*.

There were significant two-way interactions between all three factors relating to surface features: *Figuration*, *Activity*, and *Consistency*. A spreading interaction between *Figuration* and *Activity* (*F*_1,81_ = 11.85, *p* < .001, $\eta_{p}^{2}$ = .128) showed highest ratings for conditions in which both figuration and activity were present (F_1_A_1_; absence of surface feature consistency). A significant spreading interaction between *Figuration* and *Consistency* (*F*_1,81_ = 9.54, *p* < .01, $\eta_{p}^{2}$ = .105) showed a similar trend whereby ratings were highest for conditions in which figuration was the same in both nonadjacent sections (Figure 10b). Finally, there was a significant crossover interaction between *Activity* and *Consistency* (*F*_1,81_ = 8.53, *p* < .01, $\eta_{p}^{2}$ = .095). This interaction had more variance than the others (see Figure 10c). However, the trend remained: ratings were higher when activity was present and consistent between nonadjacent sections.

There were no two-way interactions between *Nonadjacent key relationship* and any other factor. There was a significant three-way interaction between *Nonadjacent key relationship*, *Figuration*, and *Consistency* (*F*_1,81_ = 4.52, *p* < .05, $\eta_{p}^{2}$ = .053), suggesting that, as hypothesized, relationships between factors play a more complex role in memory for key that can be determined from main effects.

**Figure 11.**
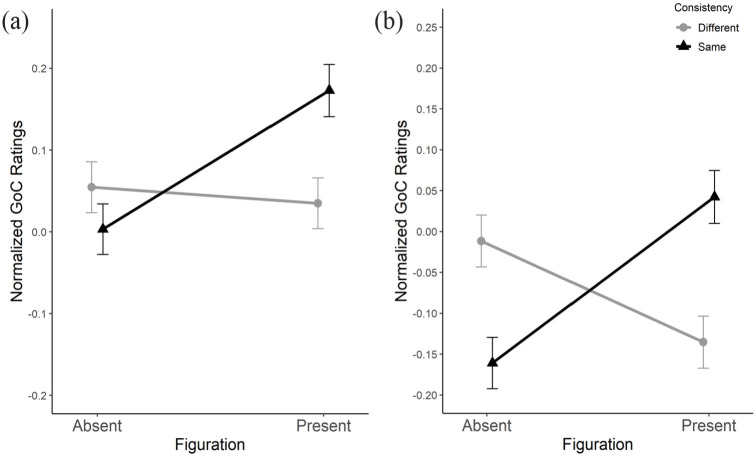
Means and standard error for *Nonadjacent key relationship*, *Figuration*, and *Consistency* in a three-way interaction showing that congruent key relationships (a) were rated higher for goodness of completion (GoC) than incongruent key relationships (b) and highest of all when the *Nonadjacent key relationship w*as congruent (a), and there was consistent *Figuration*.

For clarity, three-way interactions are shown by illustrating one of the main factors, in this case *Nonadjacent key relationship*, in separate graphs for each of its levels (i.e., congruent and incongruent). Each graph shows pairs of interactions between the remaining two factors, *Figuration* and *Consistency*, illustrated as separate lines with error bars. For example, Figure 11a shows the interaction between *Figuration* and *Consistency* when *Nonadjacent key relationship* is congruent. Figure 11b shows the same interaction when *Nonadjacent key relationship* is incongruent. As expected, congruent relationships between the keys of nonadjacent sections (Figure 11a) were rated higher than incongruent relationships (Figure 11b), clearly demonstrating the main effect of *Nonadjacent key relationship* as reported above. The relationship between *Figuration* and *Consistency* in the congruent condition (Figure 11a) provides a more detailed understanding of memory for key, however. When *Figuration* is absent, there is no difference between levels of *Consistency* (same and different). The mean for different *Consistency* but present *Figuration* remains similar to means when *Figuration* is absent. It is only when *Figuration* is present and *Consistency* is the same between nonadjacent sections that ratings become significantly higher. In fact, this specific condition in Figure 11a yielded the highest ratings of all other factor combinations. This implies that, in addition to the increase of goodness of completion ratings in conditions with congruent key relationships, there is also a significant boost when a consistent surface feature is present, in turn suggesting that memory for harmonic key is, indeed, increased by the presence of figuration.

As in Experiment 1, *Musician* was included post hoc in a repeated-measures ANOVA that yielded no interactions with the other factors. Again, control factors were not included in the main analysis.

### Discussion

Participants with and without musical training reliably perceived nonadjacent key relationships and could remember harmonic information despite intervening musical sequences. We were surprised not to find significant main effects of either *Activity* or *Consistency*, given that they were found in Experiment 1. It may be that collecting data online because of the Covid-19 pandemic introduced undue noise to the results; this could be verified in future by running the same experiment in person.

The significant two-way interactions between *Figuration*, *Activity*, and *Consistency* support the results of Experiment 1: closing probe cadences were rated higher for goodness of completion in the presence of surface features, as were nonadjacent sections when surface features were consistent. The significant three-way interaction between *Nonadjacent key relationship*, *Figuration*, and *Consistency* based on higher ratings for congruent key relationships when figuration is present and consistent suggests that surface features do indeed play a role in memory for key. In summary, the results of Experiment 2 largely support and, through the inclusion of nonadjacent harmonic relationships, extend the findings of Experiment 1 and the research of Spyra et al. (2021), Farbood (2016), and Woolhouse et al. (2016).

## General discussion

This study supports the hypothesis that surface features increase the perception of structural cohesion in music. The presence of *Figuration, Activity*, and *Consistency* in the stimuli produced higher ratings for goodness of completion in Experiment 1. Although the main effects of *Activity* and *Consistency* did not reach significance in Experiment 2, there was a significant interaction between them, suggesting these factors are more dependent on each other than previously thought. Congruent *Nonadjacent key relationship* also produced higher ratings for goodness of completion, supporting the hypothesis that relationships between nonadjacent keys are perceived as such by the listener. Furthermore, a significant three-way interaction between *Nonadjacent key relationship*, *Figuration*, and *Consistency* supports the main hypothesis that the addition of surface features may enhance memory for key either directly or through a cumulative effect on the listener’s sense of completion. More studies are needed to provide further evidence to support these suggestions.

From the perspective of music analysis, these findings could be considered counterintuitive: many analytical approaches to music proceed by removing surface features until a background structure (or *Ursatz*) is revealed (Pankhurst, 2008, pp. 54–55; Schenker, 1906/1954, p. 43); see also Bharucha (1994) and Lerdahl and Jackendoff (1983/1996). While analysts such as Heinrich Schenker did not claim that surface features are unimportant, the stripping away of melody and rhythm implies a hierarchical relationship between elements; without a well-formed (contrapuntal) background structure, the surface features of a piece of music will be perceived as somehow inferior (Schenker’s idealized conception of so-called organic composition). While our results do not refute this proposition, they do suggest that surface features play a central role in the perception of musical structure, at least over the duration of our stimuli. Moreover, a significant effect of the musical surface was found from post hoc analysis, irrespective of the harmonic sequence employed: *Figuration* and *Activity* were significant, *Sequence* was not.

We expected to find significant differences between musicians and nonmusicians but did not do so. Previous research suggests that musicians and nonmusicians pay attention to different aspects of music. For example, Tan and Spackman (2005) found that nonmusicians focus more on surface features, while musicians attend to the structure and content of pieces. Such a difference was not found in either of the experiments described above. However, there is a possibility that the participants in Tan and Spackman’s study (2005; also Experiment 1 of Deliège et al., 1996) may have detected similar differences, but the nonmusicians lacked the language to describe them as the musicians did (see also Eitan & Granot, 2008; Tillmann & Bigand, 1996). Like McAdams (1989), Tan and Spackman note that deeper structures are often reflected in the musical surface, suggesting that even nonmusicians may detect something about the structure of a piece through surface-level changes. This view may be further supported by studies such as those conducted by Lalitte and Bigand (2006), which found that sensitivity to large-scale structures does not depend on musical ability. As stimuli lasting approximately 1 min were used in studies such as Tan and Spackman (2005), it is possible that the effect of musical training becomes apparent only when stimuli are longer than those we used. More studies must be done to understand potential differences between musicians and nonmusicians, and their nature.

The incorporation of rhythmic figurations such as suspensions and retardations could have created syncopations, giving the impression of greater rhythmic activity. Therefore, although *Figuration* and *Activity* were defined strictly and manipulated independently, the reader should be aware of a possible, albeit limited interdependence between these factors and the potential for them to be confounded. That said, a percussive timbre with a swift decay, such as piano, would have exaggerated this possible effect; we chose, instead, to employ sustained bassoon and flute timbres, which helped to distinguish the onset and offset of notes within specific voices.

Although this study explored the importance of surface features in the perception of musical nonadjacency, this research is in its relative infancy. Further development of the design is required to clarify the specific processes involved; for example, the types of figuration that contribute to the effects we observed have yet to be determined. In addition, a complete investigation of *Consistency* would be both appropriate and a natural candidate for a follow-up study, as this study constrained this factor by including only complete reversals of nonadjacent surface features. Future studies could also constrain the number of independent variables to be explored; Experiment 2 in particular included a large number of independent variables, thereby potentially underpowering the study. A follow-up experiment with fewer independent variables would improve its replicability and could provide strong support for the current findings. Finally, previous studies on nonadjacency explored effects on memory for key over time. A follow-up study could therefore be conducted to compare the effects of surface features on the limits to memory found in previous research on homophonic stimuli (e.g., Farbood, 2016; Woolhouse et al., 2016).

## Conclusion

In certain areas of music analysis, a common view is that surface features are ornamentations and therefore hierarchically less important than deeper structures or harmonic patterns. The results reported above challenge this view, however, by suggesting that surface features, rather than being mere elaborations, significantly enhance memory for key relationships and play a pivotal role in the perception of harmonic nonadjacencies. As such, this study can inform music theorists, analysts, and composers as to how the human perceptual system interprets and encodes large-scale tonal structures. If people lacked the ability to connect sonic events across time, they would hear music as a cacophony of disjointed sounds. Ideally that music should include figuration; without it, people are likely to find it hard to perceive relationships between keys.

## Notes

1. Music can be perceived at two levels. The first is local, where connections are made between single events such as temporally adjacent chords; the second is global, where connections are made between musical structures in extended sequences (Tillmann & Bigand, 2004).
